# DNA-based cell typing in menstrual effluent identifies cell type variation by sample collection method: toward noninvasive biomarker development for women’s health

**DOI:** 10.1080/15592294.2025.2453275

**Published:** 2025-01-18

**Authors:** Irma M. Vlasac, Hannah G. Stolrow, Zaneta M. Thayer, Brock C. Christensen, Luisa Rivera

**Affiliations:** a Department of Epidemiology, Geisel School of Medicine at Dartmouth, Lebanon, NH, USA; b Department of Anthropology, Dartmouth College, Hanover, NH, USA; c Department of Molecular and Systems Biology, Geisel School of Medicine at Dartmouth, Lebanon, NH, USA

**Keywords:** Epigenetics, womens health, noninvasive, menstrual effluent, biomarkers, DNA methylation

## Abstract

Menstrual effluent cell profiles have potential as noninvasive biomarkers of female reproductive and gynecological health and disease. We used DNA methylation-based cell type deconvolution (methylation cytometry) to identify cell type profiles in self-collected menstrual effluent. During the second day of their menstrual cycle, healthy participants collected menstrual effluent using a vaginal swab, menstrual cup, and pad. Immune cell proportions were highest in menstrual cup samples, and epithelial cells were highest in swab samples. Our work demonstrates the feasibility and utility of menstrual effluent cell profiling in population-level research using remotely collected samples and DNA methylation.

## Introduction

Menstruating people with access to birth control menstruate approximately 400 times during their reproductive lifespan [1], from menarche in early adolescence around 12 years of age, continuing until menopause at approximately 40–50 years of age. Menstruation is a result of a complex and highly specific system regulated hormonally through the hypothalamus, pituitary, and ovarian endocrine axis, and disruption to the menstrual cycle can be indicative of underlying health issues. As an example, irregular and long menstrual cycles are associated with a range of adverse conditions, including ovarian cancer [2] and greater risk of premature mortality [3]. Menstrual effluent is comprised of the shed uterine lining developed during the secretory phase of the menstrual cycle as well as cells from the cervix and vagina. As such, menstrual effluent may be a powerful proxy tissue for the endometrium, with recent work finding considerable overlap between menstrual and endometrial cytokine levels, cellular composition, and gene expression profiles [4,5]. Research on menstrual effluent has begun to investigate its potential as an informative biospecimen in women’s health research. Studies to date have focused on elucidating inflammatory profiles and identifying perinatal biomarkers of fertility and endometriosis [5–7], and as a determinant of adverse pregnancy outcomes.

Methylation cytometry methods can determine the cell-type admixture of biospecimens using cell reference profiles of DNA methylation and performing statistical deconvolution. Methylation cytometry methods were originally developed in whole blood and have been optimized and validated over the past few years to accurately quantify 12 cell types [8–11]. The principle of DNA-based cell typing with methylation cytometry has been extended to other tissue and specimen types including brain, skin, breast tissue, and human milk [12–14]. While methylation cytometry been applied to vaginal and cervical biospecimens [15–17], it has not been applied to menstrual effluent. To analyze menstrual effluent we applied a uterine-specific cell reference deconvolution library in the hierarchical tumor immune microenvironment epigenetic deconvolution (HiTIMED) algorithm which quantifies up to 17 cell types [18]. HiTIMED was developed for cell type deconvolution in both tumor and non-tumor normal tissues and includes 20 tissue-specific cell reference deconvolution libraries, including endometrial tissue, which is shed in menstrual effluent. Here, we demonstrate the utility and feasibility of DNA-based cell type profiling in menstrual effluent with the HiTIMED algorithm in healthy females across three collection methods: menstrual cup, menstrual pad, and vaginal swab.

## Materials and methods

### Study participants and samples

Healthy study participants (*n* = 12) were recruited through campus flyers, online communities, and in-person recruitment to a Dartmouth College Committee for Protection of Human Subjects (CPHS) IRB-approved study. Written consent was obtained, and study participants were screened based on the following eligibility criteria: 18 years of age and older, regular menstrual cycles, no current use of hormonal birth control, no history of reproductive disorders (PCOS, endometriosis, adenomyosis, gynecologic cancers), and no immunomodulatory therapy. Participants completed two questionnaires: health history, and menstrual questionnaire completed at the time of sample collection. Study kits were mailed to participants and contained supplies for menstrual pad, vaginal swab, and menstrual cup self-collection along with instructions to minimize variability among collection methods. Participants were instructed to collect samples on the second day of their period, defined as the second day following the onset of bleeding.

For swab collection, participants were instructed to insert the swab (*Puritan HydraFlock*, #25–3406-H) into their vagina and hold for 15 seconds or until completely saturated. The swab was placed in a 4 mL self-standing centrifuge tube containing 2 mL of DNA/RNA shield (*Zymo*, #R1100–250). This process was repeated twice for the collection of two swabs. To collect the pad sample, participants were provided with an organic cotton liner (natracare, #3145), with a cut 2 × 2-inch square liner secured on top. Participants were instructed to wear the liner until the square was completely saturated, which was estimated to take approximately 5 minutes but may have varied amongst participants. Once the square was saturated, using the provided tweezers, participants removed the saturated square and placed it in a 4 mL self-standing centrifuge tube containing 2 mL of DNA/RNA shield (Zymo, #R1100–250). The menstrual cup (NeoProMedical, #B092DTN2XS) was worn for sample collection after which a disposable pipette was used to aliquot 2 mL of menstrual effluent into a 4 mL self-standing centrifuge tube containing 2 mL of DNA/RNA shield (Zymo, #R1100–250). Participants were instructed to wear nitrile gloves for all collection modes and place samples in a biohazard collection bag before packaging them in the return box.

### DNA extraction and methylation analysis

DNA was extracted from menstrual cup samples using Qiagen DNeasy Blood and Tissue kit protocol, following manufacturers protocol for extraction from blood samples. DNA was extracted from menstrual pads by cutting a 1 × 1 centimeter piece of the saturated menstrual pad sample, then using it as the base material for extraction using the Qiagen DNA Investigator’s Kit. DNA was extracted from vaginal swab samples by removing the swab from the plastic swab base, then using the swab material as the base material for extraction using the Qiagen DNA Investigator’s Kit. DNA was extracted from a total of 36 samples, three samples per participant with 12 participants assessed in total. Extracted DNA samples were bisulfite modified using the EZ DNAm Kit (Zymo Research) following the manufacturers optimized protocol for Illumina Methylation arrays [19]. Bisulfite converted DNA was arrayed on the Infinium MethylationEPIC BeadChip V2 array at the Dartmouth Cancer Center Genomics and Molecular Biology Shared Resource.

### Data processing and quality control

MethylationEPICv2 Intensity data (IDATs) were preprocessed for normal exponential out-of-band (Noob) correction using Minfi [20]. Quality control was performed using a detection P, CpG, and sample threshold of 5%, with zero samples demonstrating a percentage of low-quality CpG values greater than 5%. Beta value technical replicates were collapsed by averaging probes with common probe ID prefixes using SeSAMe [21]. Single nucleotide polymorphisms (SNPs), probes were used as a quality control metric to assess concordance amongst sample type and participant. Non-CpG cytosine methylation, cancer somatic mutation probes, and probes located on sex chromosomes were filtered. Beta values were normalized using beta mixture quantile normalization (BMIQ). For quality control, sample matching was confirmed using SNP probes.

### Statistical analysis

Cell proportions were estimated using Hierarchical Immune Tumor Deconvolution (HiTIMED) [18], designated at deconvolution layer five, set to non-tumor normal tissue with the uterine carcinoma deconvolution library. Cell proportions from each sample type (menstrual pad, menstrual cup, vaginal swab) were assessed for significant differences between the three collection sample types using a Friedman non-parametric test. Unsupervised hierarchical clustering was performed using the top 20,000 most variable CpGs. A Recursively Partitioned Mixture Model (RPMM) [22] was fit using the 20,000 most variably methylated CpGs to identify subgroups within the methylation data. Association of RPMM cluster membership with sample collection method was tested with a Fisher’s exact test. RPMM cluster membership by sample type uses a Binary Latent Class Tree (blcTree) hierarchy for clustering. Additional cell deconvolution methods were applied to assess cell estimates using default parameters; MethylResolver [23], EpiDISH [24] and HEpiDISH using the FlowSorted.Blood.EPIC six immune cell library [10], with differences in each collection sample type assessed using a Friedman non-parametric test.

## Results

**Figure 1.**
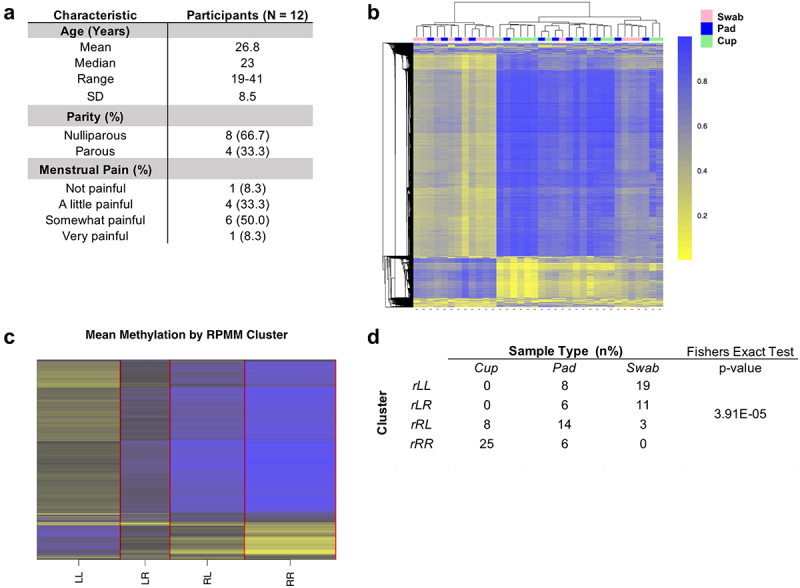
a) Study participant data. b) Unsupervised hierarchical clustering based on top 20,000 most variable CpGs. c) Recursively partitioned mixture model (RPMM) with mean methylation displayed in each of the four clusters, where the mean methylation value is demonstrated by color (yellow = 1.0, black = 0.5, blue = 0.0). d) table of RPMM cluster membership using binary latent class tree (blctree) hierarchical clustering. Cluster abbreviations indicate the position of the cluster in the hierarchy as follows: rLL (**L**eft child of the **L**eft child of the **r**oot); rLR (**R**ight child of the **L**eft child of the **r**oot); rRL (**L**eft child of the **R**ight child of the **r**oot); rRR (**R**ight child of the **R**ight child of the **r**oot).

The 20,000 most variably methylated CpGs across DNA methylation data from each collection method in twelve participants (Figure 1a) was visualized with unsupervised hierarchical clustering (Figure 1b). A recursively partitioned mixture model (RPMM) grouped samples into four methylation classes (Figure 1c), and sample collection method was significantly associated with methylation class membership (Fisher’s Exact *p* = 3.9E–05, Figure 1d).

**Figure 2.**
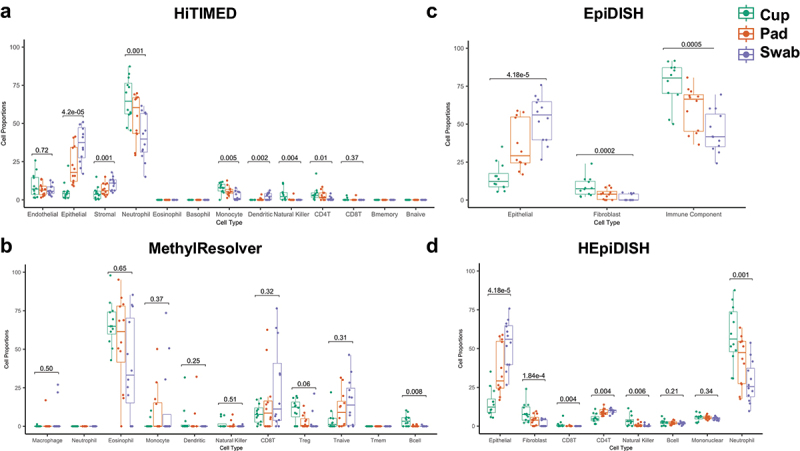
Application of DNA methylation-based cell deconvolution algorithms to bulk DNA methylation from menstrual effluent. a) HiTIMED deconvolution using deconvolution layer five and uterine reference; b) MethylResolver cell deconvolution of menstrual effluent samples; c) EpiDISH deconvolution of menstrual effluent; d) hiearchical EpiDISH (HEpiDISH) using the FlowSorted.Blood.EPIC six immune cell type reference library.

Menstrual effluent cell type proportions were estimated using HiTIMED in all samples and compared among collection methods. We selected the HiTIMED cell deconvolution algorithm because it allows specification of a uterine reference deconvolution library, which may be more robust for cell deconvolution considering menstrual effluent may serve as a proxy for the endometrium. HiTIMED epithelial and stromal cell proportions were significantly higher in swab samples compared to pad samples, with cup samples demonstrating the lowest epithelial, stromal cell proportions and highest dendritic cell proportions; cup samples had significantly higher neutrophil, monocyte, natural killer, and CD4T cell proportions compared to swab samples (*p* < 0.05, Friedman test, Figure 2a). In addition, although not all methods resolve as many or all the same cell types as HiTIMED, we compared cell proportion estimates from HiTIMED to those from MethylResolver and EpiDISH deconvolution methods (Figure 2). MethylResolver demonstrated significantly higher B cell proportions in cup samples compared to both pad and swab samples (Figure 2b), whereas HiTIMED did not identify the presence of B cells, and HEpiDISH identified B cells but demonstrated no significant difference in B cells among the three collection methods. Among granulocytes MethylResolver only identified presence of eosinophils, whereas HiTIMED identified neutrophils. EpiDISH, which estimates three cell lineages, demonstrated significant differences across all three collection types, with cup and pad samples having greater immune and fibroblast proportions compared to swab samples, and swab samples having significantly higher proportions of epithelial cells (*p* < 0.05, Friedman test, Figure 2c). Hierarchical EpiDISH (HEpiDISH) uses the same EpiDISH reference libraries but estimates immune cell component proportions using a provided immune component reference matrix. Using HEpiDISH with a six immune cell library (FlowSorted.Blood.EPIC), consistent results were observed for neutrophil, epithelial and natural killer cell proportions as with HiTIMED (Figure 2d).

## Discussion

Our work demonstrates the utility of cell type profiling in menstrual effluent with methylation cytometry for research applications in female reproductive and gynecological health and disease. Methylation cytometry cell deconvolution analysis identified cell type proportion differences by sample collection method. While EpiDISH has been applied to cervical and vaginal biospecimens in prior research, we are not aware of any studies applying methylation cytometry methods to menstrual effluent. Nené et al. investigated cervicovaginal smear DNA methylation applying EpiDISH in conjunction with assessment of the cervicovaginal microbiome to discern subtypes of microbiome using DNA methylation data [15]. In addition, Barrett et al. used methylation cytometry with EpiDISH applied to cervical smears to develop predictive approaches for ovarian cancer in high risk women [16]. Similarly, Barrett et al developed a DNA methylation based score using cervical liquid based cytology samples in women recently diagnosed with breast cancer, offering a novel method of monitoring breast cancer risk [17]. The application of Epi/HEpiDISH to menstrual effluent samples in our study yielded similar trends to HiTIMED cell proportion estimates, however, HiTIMED estimated significantly higher proportions of stromal cells in cup samples compared to pad and swab samples, whereas Epi/HEpidish estimated significantly higher fibroblast levels in cup samples compared to swab and pad samples. While HiTIMED estimates stromal cell proportions, and Epi/HEpiDISH estimates fibroblast cell proportions, stromal cells are a group of cells that includes fibroblasts, so this likely indicates fibroblasts are the most abundant stromal cell type within menstrual effluent, as demonstrated using both HiTIMED and Epi/HEpiDISH. MethylResolver was developed to specifically infer leukocyte subset fractions from tumor samples, therefore use of this method is limited when applied to menstrual effluent since the algorithm won’t capture non-immune cell types, such as epithelial and stromal cells. Application of MethylResolver within our menstrual effluent samples demonstrated different patterns of cell proportions compared to both the HiTIMED and Epi/HEpiDISH algorithms, which demonstrated high neutrophil and no eosinophil cell proportions, while MethylResolver, demonstrated high eosinophil cell proportions and no neutrophil proportions. Considering the biology of menstruation and the endometrium, neutrophil presence increases during the premenstrual phase and localize to regions of endometrial tissue degradation, responding to inflammation and likely participating in destruction of the functionalis tissue layer of the endometrium [25,26]. Additionally, mouse models have demonstrated that the presence of neutrophils are required for endometrial repair during menstruation, and eosinophils were rarely observed during endometrial tissue breakdown or repair, but were detectable within the myometrium layer of the endometrium [27]. While there are some limitations to each deconvolution algorithm assessed within this study when applied to menstrual effluent, we used the HiTIMED cell proportions for our overall interpretation of our findings, because it captures both immune and non-immune cell compartments and was specified to use a uterine reference when applied to our menstrual effluent samples.

Menstruation is a process of tissue inflammation and is marked by an increase in myeloid immune cells and leukocytes recruited in response to inflammation from necrotizing and shedding tissue [28,29]. Using the HiTIMED cell proportions, we observed menstrual cup samples had the highest immune cell proportions, specifically neutrophil, monocyte, natural killer, and CD4T cell proportions in comparison to menstrual pad and vaginal swab samples. Considering diseases marked by abnormally high inflammation, such as endometriosis, future studies utilizing menstrual effluent should consider collection method as it relates with the study goals and hypotheses. Studies aimed at elucidating immune cell profiles with gynecologic health and disease may be better powered to discern effects with menstrual cup and pad collection methods. Similarly, studies which may be more focused on assessing conditions that affect epithelial and stromal cells of the cervix or vagina, may favor menstrual pads or vaginal swabs for sample collection. It is important to recognize that cell type pattern variation may potentially be due to biological variation naturally occurring within the samples and is an important consideration for studies implementing menstrual effluent collection. While research assessing the inter and intra-personal variability of menstrual effluent remains to be assessed, our work demonstrates feasibility of menstrual effluent as a biospecimen, with the application of methylation cytometry methods enabling investigation of cell-type-dependent and cell-type-independent associations with health and disease.

We expected cell type proportion differences based on collection method similarly to how we observed them. The vaginal swab collection method had higher epithelial cell proportions, which is likely related to the method of sample collection considering the participant collected the sample shallowly within the vaginal canal as opposed to closer to the cervix. Similarly, the menstrual pad collection likely demonstrates higher epithelial cell proportions considering the shed menstrual effluent travels through the vaginal canal and vulva before being absorbed on the menstrual pad. While menstrual cup sample collections demonstrate the highest immune cell proportions compared to other sample collection methods, menstrual effluent collected by menstrual pad holds great promise for future research studies given it captures cell types from the immune, epithelial, and stromal cell compartments. Considering menstrual pad collection is the only method of collection that doesn’t require insertion, it allows for a truly non-invasive, non-penetrative sample collection, allowing for sample collection from adolescents and individuals that may not want to use an insertional collection method. As menstrual pads can easily and inexpensively be shipped in the mail and are widely available, they may provide greater potential in the scope of population level studies and studies conducted in resource limited populations. Use of DNA-based cell typing in menstrual effluent samples combined with remote, noninvasive sampling methods potentiate both research and future clinical applications of menstrual effluent specimens that benefit women’s health.

## Supplementary Material

#Supplementary Table 1.docx

## Data Availability

Data can be accessed through the Gene Expression Omnibus (https://www.ncbi.nlm.nih.gov/geo/query/acc.cgi?acc=GSE275888) and will be made publicly available upon publishing of this manuscript.
